# A Case

**Published:** 1838-01-03

**Authors:** W. Pepper


### A CASE

*Of severe inflammation of the stomach and intestines, produced by pork, with autopsy and remarks.*

BY W. PEPPER, M. D.

Mrs. W. aged 50 years, of a robust frame, and in the enjoyment of general good health, was seized on the evening of the 29th November with severe pains in the stomach, followed by vomiting.

It appears that previous to her attack her bowels had been constipated for several days, and she believed herself to have bilious colic to which she had been subject. The medical gentleman first consulted in her case gave opiates, with the view of relieving the spasms and vomiting, which had been for several hours extremely violent. Nothing however could be kept in her stomach, and small doses of calomel and rhubarb were then given to move her bowels, but without effect. Hot fomentations and sinapisms were applied to the abdomen, and injections were also resorted to with the same want of success. She came under my care on the morning of 1st December, 10 A. M. Her symptoms were greatly aggravated. The pains at first seated in the stomach, intermittent, and relieved by pressure, now became permanent and extended throughout the abdomen. Pulse 140, extremely small and intermittent,—face and extremities cold and covered with perspiration; respiration short, anxious, and performed by the intercostal muscles. Abdomen tympanitic and very tender—tongue moist and clean—great thirst—slight delirium. I was informed by the nurse that the pains and vomiting had continued during the previous night, and that the patient had also been much troubled with tenesmus, and frequent small evacuations of bloody mucus, mixed with black offensive matter.—She had occasional syncopes. As the patient was now approaching a state of perfect collapse, she was directed to take a tablespoonful of cold milk punch every fifteen minutes—calomel gr. v. and pulv. opii gr. ss every hour—a blister 6 by 8 over her stomach and sinapisms to her extremities.

3, P. M. Vomiting and purging had ceased for the last three hours. No pulse could be felt in the radial artery, and the pulsations were feeble and at least 150 in the brachial artery. Extremities cold and purple—much jactitation, with constant moaning—abdomen greatly distended—body covered with a clammy sweat—hot stimulating frictions to surface—injection of brandy and water—gr. i sulph. morphia applied to vesicated surface over stomach. I saw her again at 8 P. M. and found that she had vomited all the fluids taken and mixed with much gastric mucus. She was now in a state of perfect collapse, and expired the same night at 12 o'clock.

Autopsy 20 hours after death—present, Dr. Kirkbride—skin of extremities and posterior part of body of a livid colour. No emaciation—no œdema—abdomen enormously distended.

The mucous membrane of the large extremity of the stomach, was minutely injected and thickened. A number of red spots about a half inch in diameter were seen on different parts of the same membrane. Large peices of pulpy macerated flesh were found in the stomach, and a small quantity of white powder was seen adhering to its mucous membrane. The mucous membrane of duodenum and of upper part of jejunum had occasional small patches of inflammation. The rest of the small intestine was perfectly purple—mucous coat minutely injected and thickened—cellular and muscular coats greatly congested. The peritoneal coat was of a dark purple colour—glands of Brunner and Peyer enlarged. The small intestine contained a large quantity of black offensive fœcal matter.

The large intestine was much distended with gas. Its mucous coat was also injected and a considerable quantity of black liquid fœces were found in the colon.

The mesentery was much thickened and turgid with blood; about ℥viii of bloody serosity in the cavity of the peritoneum. No alteration or perforation, nor anykind of obstruction existed in the intestines.

The other organs were perfectly healthy.

I neglected to mention that some yellow fœcal matter was found in the duodenum and stomach.

REMARKS.

The symptoms at the onset of this disease, somewhat resembled perforation of the intestines, strangulated hernia, or *intus susception;* and in the 2nd stage, when the bloody dejections commenced, they greatly resembled the symptoms produced by an excessive dose of arsenic. During her life it could not be ascertained that the patent had taken any improper or indigestible food; but previous to the autopsy, I was informed that she had dined and supped on fat pork, which had been killed on the day of her attack. In No. CXXIX of the Edinburgh Medical and Surgical Journal, are observations on the deleterious effects of pork, contained in a letter to Professor Christison, by John M. Divitt, M. D From these observations it would appear that fresh pork, in peculiar conditions of the intestinal canal is capable of producing symptoms of the most violent character, and in most respects similar to those above recorded. I have seen several cases of cholera morbus produced by the same food; but the symptoms were comparatively mild. The only treatment calculated to relieve the symptoms, is the removal of the offending matter by a prompt emetic or cathartic. In this case pieces of pork swallowed by the patient, when macerated in the fluids of the stomach, were of such a size that it became impossible for them to escape from that organ. The excessive irritability of the stomach, rejecting even the most bland fluids, prevented the administration of such remedies as might have been proper under different circumstances. Dr. Divitt mentions that he had not seen a single case prove fatal; but believes that such might be the result under peculiar circumstances.

This case confirms the above opinion, and shows that pork may produce such intense *gastro enteritis* as to cause death in little more than 48 hours.

The stomach, with its contents was carefully removed and subjected to *chemical analysis* by Dr. Bridges. The white powder adhering to its mucous membrane, proved to be calomel, and no traces of arsenic or any other poison could be detected.

The severity of the symptoms, suddenness of death, the extensive inflammation detected by dissection, and the adherence of a white powder to the mucous membrane of the stomach, were circumstances sufficient to excite suspicion, and they required minute investigation. The case is therefore important in a toxicological point of view. I would refer those who feel curious upon this subject to Dr. Divitt's valuable paper.

Dec. 12, 1837.

